# Ten simple rules for surviving an interdisciplinary PhD

**DOI:** 10.1371/journal.pcbi.1005512

**Published:** 2017-05-25

**Authors:** Samuel Demharter, Nicholas Pearce, Kylie Beattie, Isabel Frost, Jinwoo Leem, Alistair Martin, Robert Oppenheimer, Cristian Regep, Tammo Rukat, Alexander Skates, Nicola Trendel, David J. Gavaghan, Charlotte M. Deane, Bernhard Knapp

**Affiliations:** 1 Doctoral Training Centre for Systems Biology, University of Oxford, Oxford, United Kingdom; 2 Doctoral Training Centre for Systems Approaches to Biomedical Science, University of Oxford, Oxford, United Kingdom; 3 Doctoral Training Centre for Life Sciences Interface, University of Oxford, Oxford, United Kingdom; 4 Doctoral Training Centre for Synthetic Biology, University of Oxford, Oxford, United Kingdom

## Introduction

Many of today’s pressing research challenges require a multifaceted approach that combines several historically distinct disciplines. As a result, there has been a surge in funding for interdisciplinary PhD programmes. Some examples include the United States National Science Foundation (NSF) Research Traineeship (NRT) [1] (succeeds the Integrative Graduate Education and Research Traineeship [IGERT] [2]); the European Research Council’s Innovative Training Network (ITN) [3]; and, in the United Kingdom, strong growth in interdisciplinary doctoral programmes across all research councils, led by the Engineering and Physical Sciences Research Council (EPSRC) and their Centres of Doctoral Training (CDTs), with the strong support of UK universities and industrial partners [4].

First and foremost, an interdisciplinary PhD is a great chance for students to pursue truly novel research, a range of different career paths, and a stimulating intellectual life. However, these benefits are often accompanied by additional academic and logistical challenges. The rules presented here aim to provide guidelines that will enable PhD candidates to maximise the benefits of interdisciplinary research whilst minimising any burdens.

The term “interdisciplinary” itself has many different meanings in common usage; for the purposes of this article, we define “interdisciplinary” as the synthesis of 2 or more disciplines, establishing a new level of discourse and integration of knowledge [5]. Whilst most of the advice for students considering interdisciplinary programmes is similar to that of traditional graduate programmes [6], there are also differences that students should be aware of and prepare for; the interdisciplinary PhD programmes described above, and interdisciplinary research in general [7], bring unique opportunities as well as challenges.

There are several different types of interdisciplinary PhD programmes, and their organisation varies widely from country to country. In the UK, interdisciplinary programmes are increasingly funded through CDTs, as mentioned above; investigation of the major funding bodies in your field is therefore a good place to start for discovering which programmes are available. Different interdisciplinary PhD programmes may also be organised very differently: courses might be very structured, including preparation courses and short rotation projects before the PhD; some might include continuous training throughout the PhD; and some may require you to teach, while others may not. Since interdisciplinary programmes are by their nature highly varied, some also allow you to start the programme without first having chosen a supervisor; this enables you to familiarise yourself with different fields before choosing a PhD topic. For this type of programme, it is less important to have a particular subject area in mind. However, if you do, it is important to investigate the research groups associated with the program before you apply to ensure that the course is compatible with your aims.

We are a group of PhD students and programme directors at the Doctoral Training Centre (DTC), University of Oxford. The Oxford DTC was founded in 2002 and has since accommodated over 550 students across 7 interdisciplinary programmes, namely: EPSRC Life Sciences Interface, EPSRC Systems Biology, EPSRC and Medical Research Council (MRC) System Approaches to Biomedical Science, EPSRC and Biotechnology and Biological Sciences Research Council (BBSRC) Synthetic Biology, EPSRC Synthesis for Biology & Medicine, EPSRC and MRC Biomedical Imaging, and BBSRC Doctoral Training Partnership. Drawing from our collective experience at the DTC, we present 10 simple rules for surviving and thriving in an interdisciplinary PhD. We highlight the importance of having a comprehensive plan with a realistic time line, maintaining good communication between all supervisors and collaborators, and making the most of the intellectual freedom that you are provided with when working in an interdisciplinary setting.

## Rule 1: Involve everyone in the planning, and make contingency plans

An interdisciplinary PhD will most likely require acquiring a wide range of new skills, involve more than one supervisor, and depend on multiple collaborators. It is therefore imperative to include all your supervisors early on when discussing and deciding the goals and aims of the PhD. Likewise, you should collectively lay out the plan to achieve those goals. This way, the expectations and requirements of everyone are more effectively managed (see Rule 2).

As with all PhDs, it is beneficial to generate a time line with specific milestones. This will allow you to assess the progress you have made throughout your PhD and identify any potential problems. Keep in mind that the PhD plan will change with time, so allow yourself room to manoeuvre. Importantly, make sure you have a contingency plan that covers eventualities such as collaborators not delivering data or dropping out entirely.

When planning your project, try to look ahead in terms of getting the right mentors and support network (see Rule 5). Ask your supervisors to make introductions; this can make it much easier to meet people, especially at the beginning of the PhD when you are still finding your feet.

Finally, ensure that you understand your administrative obligations. Depending on your institution and department, there may be different requirements for each stage of progression in a PhD. While the high degree of flexibility may be beneficial and allow you to choose the requirements that suit you best, it also means that you are more likely to fall outside of the normal procedures. As a result, the administration is not always prepared for the requirements of interdisciplinary students. It is therefore, as always, important to try to plan your last year in advance [8]. This will reduce the number of times you run into unexpected hurdles and make the final months less daunting. Take initiative and think ahead.

## Rule 2: Be a diplomat: Start managing expectations early on

Try to organise regular meetings with all your supervisors and collaborators. As well as ensuring progression of the PhD, collective planning and discussion helps to prevent frustrating situations and disagreements. Recognise that supervisors from different fields may have very different expectations for what is achievable in a particular time frame or may find it hard to judge the difficulty and likely time span of research outside their own area of expertise.

If questions regarding the direction of the PhD arise, such as the best approach to a problem, it is normally best to discuss the problem directly with all participating parties in the same room.

With several parties involved in your PhD, it is essential to keep communicating on a regular basis, with regular time slots for video conferences or face-to-face meetings.

## Rule 3: Define the boundaries of your research: Explore and familiarise, then be pragmatic

Once everyone is on the same page and you have laid out a plan for your PhD, you need to start to do your research. A “traditional” PhD student quickly develops very deep knowledge of a narrow subject area in a particular discipline, whereas an interdisciplinary student is likely to obtain knowledge that is less deep but spread out across several subject areas and multiple disciplines. It is therefore important to anticipate and explore the fields relevant to your PhD early on. Attending a wide range of seminars or even undergraduate lectures is a good way to gain a foundation of understanding in a new field and to learn discipline-specific terminology. Time spent investigating these complementary subject areas early will be beneficial in the long run, as it will enable you to see the bigger picture and place your work in context.

Furthermore, the quality, quantity, and structure of data vary between disciplines [9]. Make sure you know what to expect, and perform “sanity checks” on the data before you use it for anything. This will enable you to identify any issues and allow you to take the necessary steps to interpret the data correctly [10].

Whilst it is important to become familiar with all the relevant topics across the disciplines of your work, you cannot learn everything. As the PhD progresses, start focusing on the core areas that are directly relevant to your research. Consider a funnel-shaped learning approach, where you learn the fundamentals of the field first and narrow down on more specialised topics at later stages. Explore and familiarise yourself, but then try to be pragmatic and goal oriented.

## Rule 4: Don’t be embarrassed: Always ask the “stupid question”

When you are exploring new fields, it is normal to feel estranged, alone, and lost. Seminars, group meetings, and research talks in this alien field can seem like they are being conducted in an entirely different language. It is not uncommon to attend meetings where unfamiliar jargon is heavily used. Consider immersing yourself and “spending time with the natives” as much as possible; this is often the quickest and most effective way to become proficient in the new field.

Being in between disciplines naturally means that a lot of time will be spent being a novice, and progress may feel slow at the beginning. You will always have to ask a lot of questions, and it is fine to solicit help shamelessly. You might feel like your questions are too simple to waste anyone’s time with, but keep in mind that most people are happy to see you engaging with their subject. Furthermore, questions from people with a different background often lead to a new perspective that might not have been considered otherwise.

Do not be afraid to ask the “stupid” question; what seems trivial might not be quite so simple.

## Rule 5: Build a network: Find other people to complement the gaps

Due to the nature of interdisciplinary research, there will always be significant gaps in your knowledge. Identify fellow researchers early on in your PhD who complement your knowledge base; you will then be able to call upon each other’s expertise when required. You might not find all the help you need in your department. A quick internet search and an email may help you to find the right group; most scientists are friendly and happy to share their knowledge. If in doubt, your supervisors should be able to put you in contact with the relevant people.

Fostering and maintaining relationships with researchers from diverse backgrounds is a key aspect of doing an interdisciplinary PhD; it will make your life a lot easier when it comes to finding someone to answer your questions, finding suitable collaborations, or getting your hands on that crucial data set. Try to find people with whom you enjoy working and who are good at what they do. It is worth spending some time on developing good relationships, as there is a good chance you will keep working together beyond the PhD. A paper specifically focusing on different aspects of cross-disciplinary collaborations is available in the 10 simple rules series [10].

When communicating with researchers in different disciplines, be sure to clarify what people mean by the terminology they use, as the same word may mean different things in different fields. As an example, consider the word “orthogonal”: in geometry, 2 lines are orthogonal if they form a right angle; in statistics, independent variables that affect a dependent variable are considered to be orthogonal if they are uncorrelated; in taxonomy, a classification is orthogonal if each item is a member of only 1 group; and in biochemistry, the 2 types of DNA base pairs are considered orthogonal interactions. Ask for and give explanations for technical jargon, as the language barrier is always present in interdisciplinary collaborations.

While it is good to have some close contacts, it is also valuable to develop a more diverse and loosely connected network. You might consider this a “dormant” network that you can dip into when the opportunity or the need arises; it may just lead to a fruitful collaboration.

Many of the most useful conversations happen when you least expect it. Try to socialise with your peers, especially in nonacademic settings.

## Rule 6: Embrace your unique skillset and use it to redefine discipline boundaries

The value of symbiotic relationships between disciplines is well recognised. It comes from the realisation that some research questions are neglected because they do not fit inside the traditional boundaries of the discipline. Similarly, suitable methods or techniques for a particular problem might already exist in another field but have gone undetected due to the isolation of disciplines. As an interdisciplinary researcher, you are well placed to tackle those neglected problems. By embracing your unique skillset and looking for opportunities to connect the dots, you may find yourself redefining traditional boundaries or facilitating a groundbreaking translation of technology from one discipline into another.

This also holds true in collaborations. Often, solutions to problems that you would consider trivial are of high value to scientists in other fields. Recognise these opportunities and be open about your competencies, even the simplest ones. You may be surprised how much you can contribute. This is a good way to gain visibility among your colleagues and open new opportunities.

However, be careful, as it is easy to become a service provider to others in a different field when you have skills that they do not. You may not want to become the IT administrator responsible for routine data processing and organisation or the lab assistant responsible for routine and tedious experiments. Only consider doing these things if they have clear limits and clear benefits.

## Rule 7: Feel free to swim against the flow, to experiment, and to fail

Established fields will commonly have a dogmatic approach to certain problems that has evolved over generations of researchers. These well-established methods have been proven to work and have a vast amount of research to back them up. However, as a researcher with a potentially different view of the problems, you may be attracted towards approaches that are considered unconventional. Do not be afraid to challenge the dogma of the field, provided your approach has previously unanticipated benefits.

Furthermore, as someone who sits between disciplines, the techniques you require may not have been developed yet or may simply never have been applied to an individual discipline before. Developing new techniques (or applying “foreign” techniques) and proving their utility comes with higher risks and potentially higher rewards. As the forerunner, you will have to figure things out for yourself. You will get stuck and your approach may fail. Do not lose motivation during these seemingly unproductive phases. You may even encounter resistance to breaking dogmas from your supervisors, so it is important to remember that you can afford to spend time on satisfying your curiosity. Exploration involves failure, but it is fundamental and necessary in science, as it results in new discoveries and ideas.

## Rule 8: Plan your career and publish accordingly

By the end of the PhD, you will have a wide set of transferrable skills. These allow you to pursue careers in niche areas as well as more general fields. Furthermore, if you identify a skill that you do not yet have, an interdisciplinary PhD is a good opportunity to build the required competencies—so start thinking early about where you want to end up. Note that there will be different requirements depending on which career path you choose to follow. There are many places your skills could take you.

Another important factor in your career will be your publication record. Different career paths may require publishing in different journals, so you need to choose carefully. Working in an interdisciplinary setting means that you have a much larger variety of journals to choose from than a single-subject PhD, where there are usually only a select few journals.

Make sure you familiarise yourself with all relevant journals. This is especially important when you publish in journals with which your main supervisor has no experience. To narrow down the search, you can discuss options with colleagues in your field(s) and choose from a number of web services and journal selectors that find suitable journals based on information from your title, abstract, and other key words. The natural place to publish will most likely be the journals whose papers you most frequently read, but also look at the journals where these papers are frequently cited. Once you have identified some candidates, consider if the aims, scope, readership, and publication history of the journal are in alignment with your manuscript. Also, familiarise yourself with the rules of the journal by reading the author guidelines, and try to identify its readership and access options. When deciding on a journal, you may not feel that it is your choice, but try to engage in the discussion with your supervisors and explain your reasoning. An interdisciplinary PhD provides many career opportunities, as your unique skill set may be appreciated in a number of different sectors. Try to shape your PhD to your career plans and aim to publish accordingly.

## Rule 9: Adjust to your audience

Interdisciplinary researchers will have to present work at meetings, seminars, and conferences with vastly different foci (e.g., to both theoretical and experimental audiences). Your thesis will probably be examined by experts from different fields. Thus, you will have to adjust your language appropriately; your audience may comprise specialists in a particular field, those with mixed backgrounds, or those with no scientific training at all. This is, of course, applicable to all researchers, but the frequency of having to change a presentation to suit an audience is increased in interdisciplinary areas. Also, remember that you may have to run your proposed presentation past multiple supervisors and collaborators, so make sure to allocate enough time for this.

For a group of mixed backgrounds or a group with a different background, it is essential that you learn to anticipate any gaps in people’s knowledge and provide a coherent story no matter how basic you think the material is. It may also be a good idea to explain concepts from several angles to accommodate as many people as possible. When describing an abstract or technical concept, avoid jargon and use visual representations where possible. Imagine explaining it to your nonexpert self before you started doing your research.

When asked to participate in science communication and public engagement events, another approach is required. A bird’s-eye view, interesting examples, and clear language are crucial for effective communication. Do not underestimate the work that goes into these presentations; you will have to think about your research from a completely different perspective to your own. When in doubt, always start explaining things in the simplest terms possible and elaborate later on, if necessary. For high-profile events or key interview presentations, always practice in front of a friendly audience and act on feedback.

## Rule 10: Relax and enjoy

A PhD is a marathon, not a sprint. Make sure you are comfortable with your project and that you are in a position to enjoy the experience. The best publication record will be of little benefit if you do not enjoy the process. Fortunately, an interdisciplinary PhD often provides unique opportunities for you to design your research around your interests. Use this flexibility and shape your work into something that you enjoy and fully embrace.

For many scientists, creativity and productivity are highest during the first days after a break. Make use of your supervisors’ and the university’s resources, engage in clubs and other activities outside of your research, and take vacations when you need them. Taking time off will benefit you in the long run, as you will return to your research with renewed energy and a fresh mind.

A PhD is a huge investment of your time, energy, and creativity—the finale of years of education and training. By following these 10 simple rules, it should be a rewarding and empowering experience for you!
